# Late regulation of immune genes and microRNAs in circulating leukocytes in a pig model of influenza A (H1N2) infection

**DOI:** 10.1038/srep21812

**Published:** 2016-02-19

**Authors:** Louise Brogaard, Peter M. H. Heegaard, Lars E. Larsen, Shila Mortensen, Michael Schlegel, Ralf Dürrwald, Kerstin Skovgaard

**Affiliations:** 1 Section for Immunology and Vaccinology, National Veterinary Institute, Technical University of Denmark, 1870 Frederiksberg C, Denmark; 2 Section for Virology, National Veterinary Institute, Technical University of Denmark, 1870 Frederiksberg C, Denmark; 3 IDT Biologika GmbH, Dessau-Rosslau, Germany

## Abstract

MicroRNAs (miRNAs) are a class of short regulatory RNA molecules which are implicated in modulating gene expression. Levels of circulating, cell-associated miRNAs in response to influenza A virus (IAV) infection has received limited attention so far. To further understand the temporal dynamics and biological implications of miRNA regulation in circulating leukocytes, we collected blood samples before and after (1, 3, and 14 days) IAV challenge of pigs. Differential expression of miRNAs and innate immune factor mRNA transcripts was analysed using RT-qPCR. A total of 20 miRNAs were regulated after IAV challenge, with the highest number of regulated miRNAs seen on day 14 after infection at which time the infection was cleared. Targets of the regulated miRNAs included genes involved in apoptosis and cell cycle regulation. Significant regulation of both miRNAs and mRNA transcripts at 14 days after challenge points to a protracted effect of IAV infection, potentially affecting the host’s ability to respond to secondary infections. In conclusion, experimental IAV infection of pigs demonstrated the dynamic nature of miRNA and mRNA regulation in circulating leukocytes during and after infection, and revealed the need for further investigation of the potential immunosuppressing effect of miRNA and innate immune signaling after IAV infection.

Influenza A virus (IAV) infections are widespread in the human population and have great impact on human health and welfare. Significant resources are linked to influenza epidemics due to excess hospitalizations and lost productivity in workplaces, as well as the need for the production of yearly updated vaccines to cover the currently circulating influenza virus strains^1^. Otherwise healthy subjects will recover within 1–2 weeks without treatment, but the infection may also lead to severe morbidity and mortality, especially in elderly and immune compromised individuals^2^. New strains of influenza virus with pandemic potential will continue to emerge due to mutation, genetic reassortment, and a complex animal reservoir.

MicroRNAs (miRNAs) are a class of short (~22 nt), endogenous regulatory RNAs that have been identified in a wide range of organisms, including animals, plants, viruses, and fungi^3^. They modulate gene expression by interfering with mRNA translation most commonly by destabilising mRNA thereby facilitating degradation. miRNA-mRNA target interactions are complex; one miRNA may target a large number of genes, and the targets of a miRNA may belong to a variety of functional groups^3^. In turn, the 3′-UTR of a single mRNA transcript may be the target for several different miRNAs, lending another layer of complexity as well as flexibility to the system. miRNA-mediated regulation of gene expression has been found to affect many cellular functions, including innate and antiviral responses, e.g. key innate immune pathogen recognition receptors (PRRs) such as Toll-like receptors (TLR) and RIG-I-like receptors (RLR) and their associated pathways^4^. Additionally, a number of miRNAs have been demonstrated to bind to influenza virus PB1 mRNA and inhibit viral replication *in vitro*^5^. Recently, locally expressed miRNAs in lung tissue have been found to be regulated in response to IAV infection in pigs, chickens, mice, and macaques^6^,^7^,^8^,^9^,^10^, but until now only three studies have investigated the role of circulating miRNAs during IAV infection^11^,^12^,^13^. These studies investigated human patients and each study employed a different type of sample material, namely whole blood^11^, peripheral blood mononuclear cells (PBMCs)^12^, and serum^13^ from human patients, respectively. It is however important to recognize that circulating miRNAs may originate from different sources; white blood cells, red blood cells, and platelets all contain miRNAs. But miRNAs are also found extracellularly in protein complexes (Argonaute, RNA-induced silencing complex (RISC)), extracellular vesicles (EVs), and associated with high-density lipoprotein^14^,^15^. The origin of cell-free circulating miRNAs is still unclear; they may be breakdown products originating from lysed cells, or they may be actively secreted from cells to act in a paracrine manner, or perhaps a combination of the two^14^. Regulation of cell-associated and cell-free miRNAs in circulation in response to IAV infection may thus have different causes and functions. In PBMCs of critically ill patients with H1N1 infection, expression of hsa-miR-29a, -31, and -148a were all determined individually to have diagnostic potential^12^, whereas the serum levels of hsa-miR-17, -20a, -106a, and -376c in combination could discriminate between avian influenza infected patients and healthy controls^13^. The third study, employing whole blood, reported some overlap with findings from the PBMC and serum studies, namely hsa-miR-29a and hsa-miR-17 and -106a, respectively^11^. Current knowledge on miRNAs in circulation in response to IAV is thus based on few studies representing both cell-free and cell-associated miRNAs.

All three studies have identified miRNAs that are regulated during IAV infection in human patients. An inherent challenge in such observational studies, however is that the timing of the infection is unknown; the infection could be widely differently progressed in the patients included. In contrast, animal models allow for precise control of parameters such as time and dose. An ideal animal model for human influenza should reproduce the clinical signs and pathogenesis observed in human influenza disease, and the host response should mimic that observed in humans, including an efficient antiviral immune response. The general potential of the pig as a large animal model for human disease including influenza has been reviewed several times^16^,^17^,^18^. Specifically, fever, fatigue, lassitude, and cough, which were the best indicators of human influenza in several studies^19^,^20^,^21^, are all induced in IAV infected pigs within the first three days of infection as shown by us and others^6^,^22^,^23^,^24^. Pigs are susceptible to infection with human influenza A viruses, and have been demonstrated to be involved in influenza evolution and ecology^18^,^25^. Pigs additionally share many similarities with humans with respect to tracheobronchial tree structure, influenza virus receptor distribution, lung physiology, and innate immune cell infiltration of the respiratory system^26^,^27^,^28^,^29^. The pig is thus an obvious large animal model for human respiratory infections. However, the use of the mouse as an animal model for human IAV infection has been instrumental in influenza research due to its low cost, broad range of available reagents, and the availability of genetically modified mice^30^,^31^. Although this classical model has provided important information about basic biology during viral infection, mice are not natural hosts for IAV and many important clinical signs characteristic of human influenza virus infection are absent in mice^31^. The ferret model has also contributed substantially to the understanding of e.g. IAV pathogenicity, and transmission as well as species and cell tropism, in large part thanks to the ferret being susceptible to human IAV strains^30^. However, little is known about ferret specific innate immune response to IAV infection^30^,^31^. Table 1 summarizes some of the most important similarities and differences between man, pig, and mouse with regards to their clinical and immunological responses to IAV infection.

Despite the great potential of the pig as a large animal model and the importance of pigs in evolution and transmission of IAV, the immune response of pigs against IAV is not fully understood. This study aimed at providing a better understanding of the involvement of circulating miRNAs and innate immune factors in porcine blood leukocytes during and after active IAV infection. We focused on the cell-associated miRNA and mRNA expression. Cells in the lungs produce chemokines in response to IAV infection which direct the recruitment of various immune cells from the blood stream into the infected lung tissue^6^,^32^. miRNA expression profiles of circulating leukocytes may thus be important for immune regulation in the lung during IAV-infection, or even during subsequent secondary infections. Our experimental setup allowed us to perform highly controlled experimental IAV infection of pigs, and to study development of disease signs, viral titre, and fluctuations in miRNA and mRNA levels as the disease progressed in time based on a statistically useful number of pigs, generating important information relevant for human disease, which would be difficult to obtain from human patients.

## Results

### Infection model and clinical signs

Clinical signs and results from virus specific qPCR from lung samples and nasal swabs of infected animals have been reported previously^6^. This included abrupt onset of disease with dyspnoea, fatigue, nasal secretion and nasal viral excretion on day 1 pi. Fever >40 °C was seen for all but one pig (39.6 °C) on day 1 pi. Clinical signs peaked between day 1 and 2 pi. Between day 3 and 7 pi coughing occurred in individual pigs. The infection and disease were completely cleared at day 14. Lung virus titres peaked at day 1 pi; mean log_10_ EID_50_/g lung tissue was 4.23 and 3.35 at day 1 and 3 pi, respectively. No virus could be detected in the lungs by day 14 pi.

### Quantification of leukocyte miRNAs in circulation

An initial screening for changes in miRNA expression in leukocytes at 24 and 72 h pi compared to before challenge was performed using the miRCURY LNA Human Panel I (Exiqon) assaying 375 human miRNAs. Of these, approx. one third was quantifiable in porcine leukocytes; however, only five were significantly differentially expressed (one-way ANOVA): hsa-miR-223-5p (*p* = 3.20E-07), hsa-miR-23a-3p (*p* = 0.0001), hsa-miR-30c-5p (*p* = 1.10E-07), hsa-miR-150-5p (*p* = 0.0002), and hsa-miR-92b-3p (*p* = 0.0005).

Results from the miRCURY LNA screening revealed the need for a more tailored approach; relevant miRNAs were selected for expression analysis in a larger set of samples, using the high-throughput RT-qPCR platform BioMark (Fluidigm). This selection included several of the miRNAs assayed in the miRCURY LNA Human Panel I, including four of the five miRNAs that were significant on the miRCURY platform. Using Student’s *t*-test or Mann-Whitney *U* test (*p* < 0.05) to determine statistical significance of biologically relevant alterations in expression level (> ±1.5 fold up- or down-regulation) showed that results obtained from the two qPCR platforms were in agreement (data not shown).

**Figure 1:**
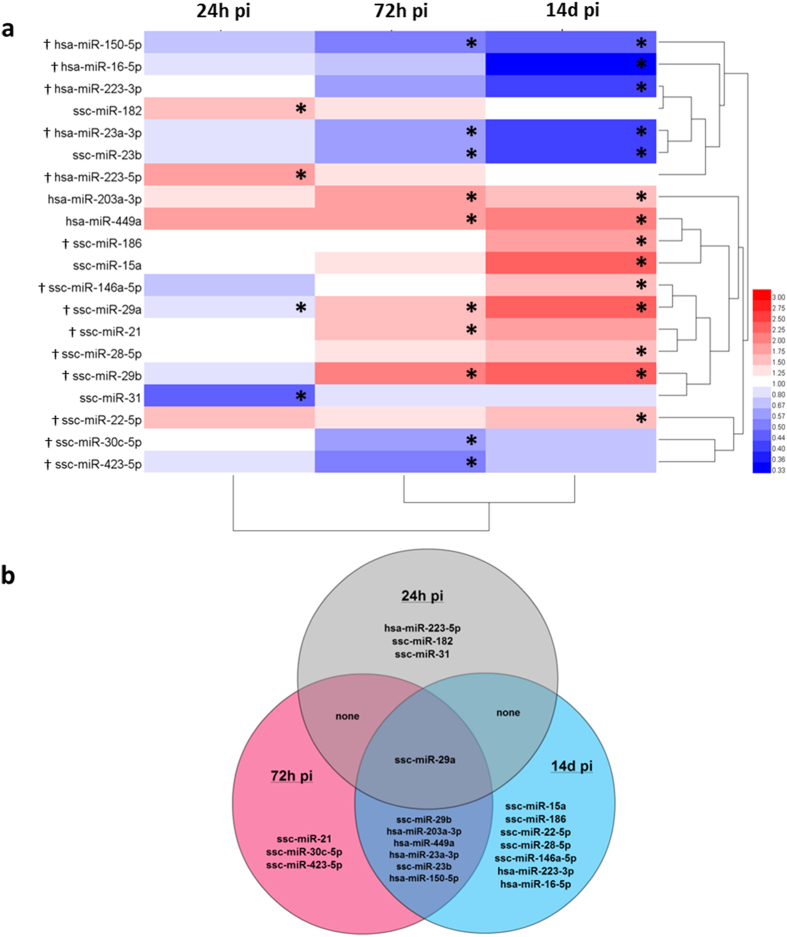
Expression of miRNA in porcine leukocytes after IAV challenge. (**a**) clustered heat map depicting up- and down-regulation (red and blue, respectively) at 24 h, 72 h, and 14d pi compared to before challenge. *Indicates statistical significance at that time point. ^†^Indicates that the miRNA have previously been reported to be regulated in circulation in human patients after IAV infection^11^,^12^,^13^. Human (hsa-) miRNAs: hsa-miR-223-3p, hsa-miR-223-5p, hsa-miR-203a-3p, and hsa-miR-449a are not yet annotated in the porcine genome. hsa-miR-150-5p, hsa-miR-16-5p, and hsa-miR-23a-3p were not annotated in the pig genome at the time the assay was designed, but sequences have since become available and found to be 100% identical to the human homologs that were used for primer design. (**b**) Venn diagram showing at which post challenge time points miRNAs were regulated compared to before challenge.

Using high-throughput RT-qPCR we found a total of 20 miRNAs to be differentially expressed in IAV challenged pigs at minimum one of the three post-challenge sampling points compared to before challenge as depicted in the heat map in Fig. 1a; the precise expression levels relative to before challenge can be found in Supplementary Table S3. Only four miRNAs (hsa-miR-223-5p, ssc-miR-31, ssc-miR-29a, and ssc-miR-182) were differentially expressed at 24 h pi, whereas 10 miRNAs were differentially expressed at 72 h pi (ssc-miR-29b, hsa-miR-203a-3p, hsa-miR-449a, ssc-miR-21, ssc-miR-29a, hsa-miR-23a-3p, ssc-miR-23b, ssc-miR-30c-5p, ssc-miR-423-5p, and hsa-miR-150-5p). However, the highest number of significantly regulated miRNAs compared to before challenge was found at 14d pi, at which time point the infection had completely cleared (ssc-miR-15a, ssc-miR-29b, ssc-miR-29a, hsa-miR-449a, ssc-miR-186, ssc-miR-22-5p, ssc-miR-28-5p, hsa-miR-203a-3p, ssc-miR-146a-5p, hsa-miR-150-5p, ssc-miR-23b, hsa-miR-223-3p, hsa-miR-23a-3p, hsa-miR-16-5p). In all three post-challenge groups, at least three miRNAs were found to be unique for that particular time point, and only ssc-miR-29a was found to be differentially expressed at all three examined time points. Overlaps and differences in miRNA regulation at the three time points are summarised in Fig. 1b.

### Leukocyte immune gene expression

In contrast to miRNA regulation, the major changes in immune gene expression were seen at 24 h pi. Levels of expression of interferon and interferon-induced genes, pattern recognition receptors (PRRs), chemokines, and pro- and anti-inflammatory cytokines before and after infection are listed in Table 2. We were able to verify expression of genes found to be regulated in human IAV infection such as *CCL2, CXCL10* (IP-10), *MX1, OASL, STAT1, IFIH1* (MDA5), and *DDX58* (RIG-I) in our porcine model. These genes have been previously reported to be of considerable diagnostic value in human influenza studies^33^,^34^,^35^,^36^. Remarkably, all of these genes but *CCL2* were among the most highly expressed immune factors in the present study at 24 h pi (Table 2). Several RNA virus associated PRRs, including *DDX58, IFIH1, TLR7*, and *TLR8*, were regulated according to infection status, being up-regulated at 24 h pi, and showing little or negligible change in expression during the rest of the study. Surprisingly, we found the PRR *TLR4*, recognising bacterial LPS, to be the most highly up-regulated TLR at 24 h pi. The up-regulation of *IDO1* (more than 100 fold after 24 h) was also highly significant and in agreement with the overall pattern of increased expression of pro-inflammatory genes, characterised by a fast and transient up-regulation peaking at 24 h pi (Table 2). Importantly, at day 14 pi all significant differentially expressed immune genes were down-regulated; these late down-regulated genes included *CCL3, CXCL2, IL1RAP, IL18, PTSG2, TNF, IDO1, TLR3*, and *TLR4*. The most significant down-regulation was seen for *IL18* at 24 h pi. This was also the only gene that was significantly down-regulated at all three time points.

### Identification of targets of regulated leukocyte miRNA

KEGG pathway enrichment analysis of the validated gene targets of the 20 differentially expressed miRNAs revealed a big overlap in pathways enriched in the four subsets of target genes. Significantly enriched “p53 signaling”, “Cell cycle”, “Apoptosis”, as well as pathways related to immune response and leukocyte extravasation, e.g. “Cytokine-cytokine receptor signaling”, “Chemokine signaling”, “Jak-STAT signaling”, “Focal adhesion”, “Adherens junction”, and “Tight junction”. In addition, several cancer related pathways were identified as significantly enriched as well, possibly due to many of the gene targets being involved in apoptotic and anti-apoptotic processes, as will be discussed later. A list of significantly enriched pathways can be found in Supplementary Table S4.

Differentially expressed miRNAs (human homologs) and experimentally validated MTIs (obtained from miRTarBase) were submitted to Cytoscape, creating interaction networks for all three post-infection time points. Of the 20 examined miRNAs, only two (hsa-miR-223-5p and hsa-miR-22-5p) did not have any MTIs registered in miRTarBase. At all three time point, we found a large number of genes to be targeted by only one of the regulated miRNAs (see Supplementary Fig. S1), but also genes targeted by two or more miRNAs were present in the interaction networks at all three time points. The number of genes with two or more experimentally validated MTIs comprised 6, 65, and 84 at 24 h, 72 h, and 14d pi, respectively (see Supplementary Fig. S1).

### miRNA target gene expression

Following MTI analysis, a subset of genes found to be targets for regulated miRNAs were subjected to transcriptional analysis. A complete list of genes can be found in Supplementary Table S1. The following genes were found to be significantly (however less than 2-fold) up- or down-regulated compared to before challenge: at 24 h pi – *BCL2, MCL1*, and *SP1* (up-regulated), *IFNG, PTEN* and *CXCR4* (down-regulated); at 72 h pi – *VEGFA* (up-regulated), *CCNE2* (down-regulated); at 14d pi – *VEGFA* (up-regulated); *CCNE2* and *PTEN* (down-regulated). Additionally, the following genes showed no significant differential expression: *CDK2, CDK4, FOXO3A, TP53, CASP9, CHUK,* and *AKT2*.

## Discussion

Similarities and differences between human, pig, and mouse with regard to influenza and factors of importance for development of influenza are highlighted in Table 1. It is evident based on both physiological and immunological considerations, that the porcine model has the potential to provide valuable information in the study of IAV infection^18^,^28^. We did indeed observe clinical signs in the animals post challenge highly similar to those observed during human IAV infection^6^. We were able to reproduce key findings of human coding and non-coding transcriptional responses to IAV infection, consolidating the pig as a relevant large animal model for the study of IAV infection in controlled settings.

Early cytokine responses to IAV infection have been found to be remarkably similar between humans and pigs^6^,^20^,^23^,^24^. In the present study we found blood-based gene expression profiles of both coding and non-coding RNA to be tightly regulated in accordance with progression of infection and disease in pigs experimentally infected with the H1N2 influenza virus. Expression of several protein coding genes in whole-blood RNA from human patients or volunteers have been found to be good predictors for IAV infection^33^,^34^,^35^,^36^,^37^. These genes include *CXCL10* (IP-10), *MX1, OASL, STAT1, IFIH1* (MDA5), and *DDX58* (RIG-I), and *CCL2*, which we also found to be highly expressed shortly after experimental IAV infection in pigs. Likewise, the expression of porcine interferon (*IFNA1* and *IFNG*) and interferon-related genes after viral infection were highly similar to those seen in human influenza cases^35^,^36^,^37^.

We and others have reported locally produced miRNAs in lung tissue to be differentially expressed in response to IAV infection in a number of different animal models^6^,^7^,^8^,^9^,^10^. However, expression signatures of miRNAs in circulation after IAV infection have only been reported in a few studies^11^,^12^,^13^. These studies have employed whole blood, PBMCs, or serum supplied by hospitalised patients and healthy volunteers and the results therefore represent the circulating miRNA response to IAV infection collected from individuals that may have had widely differently progressed disease, and from blood fractions that may carry very different miRNA profiles even before IAV infection. However, 13 of the 20 differentially expressed miRNAs identified in the present study have been reported in the abovementioned human studies as relevant for distinguishing between healthy controls and IAV infected patients^11^,^12^ (Fig. 1a). Almost complete sequence conservation is seen between human and porcine homologs of these miRNA, also within the important seed sequences (Supplementary Table S2). It is therefore reasonable to expect these conserved miRNAs to have the same biological function in response to IAV infection in pigs and humans.

Our experimental setup proved useful for demonstration of the temporal dynamics of miRNA expression after IAV challenge, from the first days of infection to after the IAV infection had cleared. In contrast to the human studies, the pig model allowed us to demonstrate that there is indeed a time factor to consider when assessing the regulation and involvement of cell-associated circulating miRNAs in response to IAV infection. This is made evident by our observations of e.g. ssc-miR-29a expression; this miRNA is initially down-regulated (24 h pi) but later up-regulated (72 h and 14d pi) (Fig. 1a), whereas human studies have reported only down-regulation of its homolog hsa-miR-29a-3p^11^,^12^,^13^. Another miRNA identified in human studies as regulated after H1N1 infection is hsa-miR-150-5p which was reported by one study to be up-regulated^11^ while it was found to be down-regulated in another study^12^. We found that hsa-miR-150-5p was down-regulated in porcine leukocytes 72 h and 14d after H1N2 challenge in pigs (Fig. 1a). Many factors other than host species may contribute to these differences, e.g. the lack of control of the infection progression in human patient studies, patient heterogeneity, difference in IAV subtypes, and the application of different blood components in the analyses. Still, time course studies of miRNA expression in controlled experimental settings using standardised sampling methods are able to provide important data when studying the role of miRNAs in circulation in response to infection and in the search for valid biomarkers.

Genes for several well-known PRRs and pro-inflammatory factors were significantly down-regulated at day 14 pi, after the viral infection had cleared (Table 2). These data suggest a persisting lowered immune responsiveness in the animal. Critical illness and severe sepsis have previously been shown to leave patients in a protracted immunosuppressed stage after the illness has passed^38^, but here we show that even a mild course of influenza disease may transcriptionally affect the immune competence of the recovered animal. Likewise, expression of miRNA at day 14 pi with the potential to subtly affect and regulate important apoptosis and cell survival related processes may have an essential function in the case of secondary infections following IAV infection. Upon secondary infection, circulating leukocytes will be recruited to the site of infection, and their built-in arsenal of apoptosis-regulating miRNAs could thus greatly influence disease progression.

Studies of secondary bacterial infections in mice after IAV infection have demonstrated a desensitisation to TLR ligands even several months after the IAV had cleared^39^. This appears to be a strategy for avoiding excessive inflammation, but the consequence of this lowered responsiveness may also be increased susceptibility to secondary infections. Here we show that ssc-miR-146a-5p is significantly up-regulated in circulating leukocytes only at 14d pi; the human homolog of this miRNA has been found to target important components of TLR signaling, such as *TLR2, TLR4, NFKB1, IRAK1, TRAF6*, and *IL8*. hsa-miR-203a-3p, which targets *MYD88*, is also up-regulated at 14d pi. These miRNAs might contribute to the reported lack of responsiveness to bacterial TLR ligands, by inhibiting translation or increasing degradation of mRNA transcripts of key components in the TLR signaling pathway. Strikingly, we did observe that *TLR4* and *TNF* (targets of and miR-146a-5p and miR-203a-3p, respectively) were significantly down-regulated in leukocytes at 14d pi. It is also worth noting, that in contrast to most other pathways found to be enriched in our analysis of miRNA target genes, the Toll-like receptor signaling pathway was only enriched in gene targets of up-regulated miRNAs, suggesting that TLR-mediated signaling may mostly be suppressed. The long-term effect of IAV-infection on the miRNA expression profile of circulating leukocytes thus potentially affects the individual’s ability to combat secondary infections. This calls for future studies and focused investigation to elucidate if the miRNAs of circulating white blood cells truly affects the immune response to secondary infections, and offers a potential therapeutic target to avoid excess morbidity and mortality in vulnerable populations.

Both host and viral factors may contribute to the late regulation of miRNAs in circulating leukocytes at 14d pi. IAV is known to produce defective interfering (DI) virus particles during infection, i.e. virus particles that are unable to complete a full replication cycle but may be able to replicate in the host cell with the aid of a replication competent helper virus. DI particles have been demonstrated to persist in the lungs of SCID mice up to 16 days after challenge^40^. The persistence of such DI particles may explain the late regulation of miRNAs in the circulating leukocytes. Indeed, defective viral genomes (DVG) segments arising from especially the PA and PB1 gene segments have been shown to be recognised by RIG-I and may thus function as PAMPs, inducing a cytokine response (IFN-β, IL-6) through RIG-I also after the productive virus infection has vanished^41^,^42^. Although we did not observe significantly increased levels of *IFNB1* or *IL6* mRNA in the lungs of our animals at 14d pi (Skovgaard *et al*. 2013), and we have found miRNAs in the lung tissue to primarily be regulated on days 1 and 3 (Brogaard *et al*., manuscript in preparation) in contrast to on day 14 in leukocytes, we cannot rule out that DI particles have persisted in a small number of cells, influencing the lung environment and as a consequence affecting the miRNA response in circulation.

Numerous pathways are activated in the cell upon IAV infection, including the Toll-like receptor, RIG-I-like receptor, NFκB, and PI3K/AKT signaling pathways^43^, many of which are important in the induction and control of apoptosis. IAV infection induces apoptosis in host cells *in vivo* and *in vitro* and different viral proteins have been found to manipulate apoptotic signaling, including the non-structural proteins NS1 and PB1-F2^44^,^45^ and the nucleoprotein NP^46^. Several studies have shown that IAV has evolved strategies to benefit from host apoptosis, which might otherwise be considered a host defence mechanism to limit virus replication and spread. Our pathway enrichment analysis revealed that many of the same pathways may be affected in circulating leukocytes both during and after IAV infection, including pathways related to apoptosis and cell cycle regulation. These pathways were enriched in gene target subsets of both up- and down-regulated miRNA, demonstrating that miRNA regulation of these pathways is complex and allows for both down-regulation as well as expression or even up-regulation of their target genes/pathways. Consequently, only small or even non-significant changes were observed in the expression of the genes selected as highly targeted by differentially expressed miRNAs, suggesting a role for miRNAs in fine-tuning gene expression rather than causing dramatic changes.

MTI analysis of miRNAs found to be significantly regulated in porcine leukocytes revealed that several of the most highly targeted genes are relevant for apoptosis and cell survival, including *BCL2, MCL1, PTEN, AKT2, BRCA1*, and *TP53*. This suggests that miRNA play a role in regulating apoptotic pathways after IAV infection, e.g. by targeting components of some of the same pathways that are directly affected by viral protein interaction. Consistent with this emphasis on apoptosis was the fact that we found caspase-1 and caspase-3 transcription to be up-regulated within the first 72 hours of infection. The pro-apoptotic transcription factor p53 (*TP53*) has previously been implicated in IAV-induced apoptosis, mediated by direct interaction with viral NP^46^. In accordance with the importance of this transcription factor, we found miRNAs targeting *TP53* to be significantly regulated in circulating leukocytes. Other genes that were highly targeted by differentially expressed miRNAs include *PTEN* and *BCL2,* central players in cell survival and apoptosis^44^,^47^,^48^. *BCL2* was heavily targeted by up-regulated miRNAs, whereas *PTEN* targeting was balanced between up- and down-regulated miRNAs. ssc-miR-29a was the only miRNA to be regulated at all three time points. The miR-29 family is well characterised and commonly described to be involved in cancers as tumor suppressors^49^. Among the targets for hsa-miR-29a-3p is *PTEN*, which was significantly down-regulated at both 24 h and 14d pi. This is consistent with up-regulation of ssc-miR-29a at 14d pi, whereas up-regulation of ssc-miR-182 – another *PTEN*-targeting miRNA – may be a contributing factor to *PTEN* down-regulation at 24 h pi given that ssc-miR-29a is itself down-regulated at this time point.

In a previous study inhibition of miR-29a(-3p) in human hepatoma cell lines was shown to lead to up-regulation of *PTEN* at mRNA and protein levels, whereas overexpression of miR-29a down-regulated *PTEN* mRNA and protein^50^. Moreover, miR-29a inhibition negatively regulated phosphorylation of Akt (Protein Kinase B), which is an important step in the PI3K/AKT signaling pathway^50^.

Akt family member *AKT2* as well as *FOXO3A*, a pro-apoptotic transcription factor (FoxO) downstream of PTEN and Akt, are also among the targets for hsa-miR-29a-3p. Despite being heavily targeted by up-regulated miRNAs at both 72 h and 14d pi (Supplementary Fig. S1), we saw no regulation of these two genes. It is however possible that Akt and FoxO protein functions are still affected by ssc-miR-29a regulation, given the effect that down-regulation of *PTEN* is likely to have on PTEN protein levels. Additionally, we observed up-regulation of hsa-miR-29a-3p targets *BCL2* and *MCL1* at 24 h pi, where ssc-miR-29a was down-regulated. Collectively, our results highlight the importance of ssc-miR-29a in relation also to influenza, especially after infection, where it is involved in regulation of apoptosis, as has been described previously primarily in cancers^49^.

In a previous study, we investigated mRNA expression in the lung tissue of the same animals as employed in the present study^6^, and could demonstrate that the chemokine transcripts *CCL2, CCL3*, and *CXCL10* were highly up-regulated at 24 h and 72 h pi. This is in accordance with an expected need for recruitment of various immune cells to the affected lung tissue, in order to combat and contain the IAV infection. Here we show that the leukocytes available for extravasation display a miRNA expression profile specialised towards contributing to the control of cell survival and apoptosis in response to IAV infection.

## Materials and Methods

### Experimental design

This study employed blood samples from a previously described experimental challenge study^6^. All procedures and animal care activities were conducted in accordance with the guidelines and under approval of Good Clinical Practice (VICH GL9, CVMP/VICH/595/98), the Directive 2001/82/EC on the Community code relating to veterinary medicinal products and German Animal Protection Law. The protocol IDT A 03/2004 was approved by the Landesverwaltungsamt Sachsen-Anhalt, Germany (Reference Number: AZ 42502-3-401 IDT). All pigs (cross-bred Large White x German Landrace) were found seronegative for the following influenza virus strains by hemagglutination inhibition assay: A/sw/Haselünne/IDT2617/03 (H1N1), A/sw/Bakum/1832/00 (H1N2), A/sw/Bakum/IDT1769/03 (H3N2), A/sw/Denmark/13850/03 (H1N1), A/sw/Denmark/12687/03 (H1N2). A/sw/Denmark/12687/03 (H1N2) was used as challenge strain; this virus has a European avian-like H1, and a European swine-like N2^29^. This reassortant has been circulating in the Danish pig population since 2003, and has also been found in Germany, Poland, and Italy^25^. Briefly, a group of 12-week-old pigs were challenged by aerosol exposure to 6.0 l nebulised culture supernatant containing 10^4.55^ TCID_50_/ml of the challenge strain. Blood samples were collected 2–4 hours (h) before challenge (n = 12), and at three time points after challenge: 24 h post infection (pi) (n = 12), 72 h pi (n = 9), and 14 days (d) pi (n = 6). Blood samples were stabilised by heparin (HEP tubes, Terumo). Lung material was collected for virus titration at 24 h (n = 3), 72 h (n = 3), and 14d pi (n = 6) and stored in RNA*later* (Qiagen) at −20 °C.

### Clinical signs and virus detection

Clinical signs and rectal temperature were recorded morning and afternoon until 72 h pi. A composite dyspnoea score was determined based on the following clinical signs as described previously^6^: 0 = breathing unaffected; 1 = increased respiratory frequency and moderate flank movement; 2 = marked pumping breathing and severe flank movement; 3 = laboured breathing affecting the entire body, pronounced flank movement and substantial movements of the snout, 4 = severe breathing reflecting substantial lack of oxygen. Virus content of the lungs was quantified by titration in embryonated hens’ eggs on day one, three, and 14 after challenge as described previously^51^. The individual titers expressed in EID_50_/g lung tissue were determined in samples of pools from all lung lobes.

### RNA extraction

Heparin stabilised whole blood samples were depleted of red blood cells using QIAamp hypotonic EL buffer (Qiagen), followed by centrifugation at 400 × g at 4 °C for 10 min. Pellets containing leukocytes were resuspended in 600 μl RLT buffer (Qiagen) and stored at −80 °C, stabilising the RNA until extraction. For extraction, 300 μl leukocyte suspension was mixed with 1 ml Trizol (Invitrogen) and incubated for 5 min at room temperature. RNA extraction was performed according to the manufacturer’s instructions (Invitrogen). Purity of extracted total RNA was assessed using UV absorption measured on a NanoDrop ND-1000 spectrophotometer (Saveen and Werner AB); average A_260/280_ and A_260/230_ ratios were 1.87 and 1.70, respectively. Total RNA was quantified by measuring the sample absorption at 260 nm; average RNA yield was 272 ng/μl. RNA integrity was measured on an Agilent 2100 Bioanalyzer (Agilent Technologies) using the RNA 6000 Nano Kit (Agilent), yielding an average RNA integrity number (RIN) of 8.9.

### miRCURY LNA Universal RT microRNA PCR

Initial screening for differentially expressed miRNAs during the course of infection was performed on a subset of the samples from the experimentally challenged pigs: before challenge: n = 10; 24 h pi: n = 10; 72 h pi: n = 6. Screening was carried out using the Human Panel I, miRCURY LNA^TM^ (Exiqon). 20 ng total RNA was reverse transcribed in 20 μl reactions using the miRCURY LNA™ Universal RT microRNA PCR, polyadenylation, and cDNA synthesis kit (Exiqon). Negative controls (minus RT) where the reverse transcriptase was replaced with water were included to check for contaminating genomic DNA. Diluted (1:100) cDNA was assayed in 10 μl PCR reactions according to the protocol for miRCURY LNA™ Universal RT microRNA PCR (Exiqon). Each miRNA was assayed once by qPCR in the Human Panel I containing 375 miRNA assays including a non-template control (NTC). Amplification was performed in a LightCycler® 480 Real-Time PCR System (Roche) in 384 well plates. LightCycler® 480 software was used to determine the quantification cycle (C_q_) values and inspect amplification and melting curves. Data was normalised to the mean expression value of all miRNAs expressed in the individual sample.

### cDNA synthesis, pre-amplification, and qPCR of miRNA

Reverse transcription of 100 ng total RNA was performed as described previously^52^. Briefly, reaction volumes of 10 μl containing 1 μM universal RT primer (5′caggtccagtttttttttttttttvn3′), 100 ng total RNA, 1 μl of 10X poly(A) polymerase buffer (New England BioLabs), 0.1 mM of ATP (New England BioLabs), 0.1 μM of each deoxynucleotide (dATP, dCTP, dGTP, and dTTP) (Sigma-Aldrich), 100 units of MuLV reverse transcriptase (replaced with water for the minus RT control) (New England BioLabs), 1 unit of poly(A) polymerase (New England Biolabs, USA), and RNase-free water were prepared. cDNA was synthesised at 42 °C for 1 hour followed by enzyme inactivation at 95 °C for 5 min. Two replicates of cDNA synthesis were performed per RNA sample (technical replicates). All primers used in this study, including the universal RT primer, were designed using the specifications described previously^52^ and purchased from Sigma-Aldrich. Relevant miRNAs were defined based on the miRCURY LNA™ panel screening, published findings, and database searches (miRTarBase v. 4.5 and TarBase v. 6.0) for miRNAs experimentally confirmed to target antiviral immune mediators previously found to be regulated during influenza infection. Whenever an annotated porcine miRNA (“ssc-miR-“) sequence was available in miRBase, this was used for primer design. If no porcine sequence was available, the corresponding human sequence homolog (“hsa-miR-“) was used. Primer sequences are shown in Supplementary Table S1. Pre-amplification was performed using TaqMan PreAmp Master Mix (Applied Biosystems). 200 nM pooled qPCR primer mix was prepared by combining each miRNA primer pair used in the qPCR setup. 5 μl TaqMan PreAmp Master Mix, 2.5 μl 200 nM pooled primer mix, and 2.5 μl cDNA was incubated at 95 °C for 10 min followed by 18 cycles of 95 °C for 15 s and 60 °C for 4 min. Residual primers were digested by adding 16 U of Exonuclease I (New England BioLabs), and incubated at 37 °C for 30 min followed by 80 °C for 15 min. qPCR was performed in Dynamic Array Integrated Fluidic Circuit chips (Fluidigm) in the BioMark HD real-time PCR instrument (Fluidigm). Pre-sample mix was prepared using the following components per sample: 3 μl ABI TaqMan Gene Expression Master Mix (Applied Biosystems), 0.3 μl 20X DNA Binding Dye Sample Loading Reagent (Fluidigm), 0.3 μl 20X EvaGreen (Biotium, VWR – Bie & Berntsen), and 0.9 μl low EDTA TE Buffer (VWR – Bie & Berntsen). Pre-sample mix (4.5 μl) was mixed with 1.5 μl pre-amplified cDNA diluted 1:10 in low-EDTA TE-buffer (VWR – Bie & Berntsen), including a non-template control (cDNA replaced with water). Primer mix was prepared for each assay by mixing 3 μl primer pair (forward and reverse, each 10 μM) with 3 μl 2X Assay Loading Reagent (Fluidigm). The following cycling parameters were used: 2 min at 50 °C, 10 min at 95 °C, followed by 35 cycles with denaturing for 15 s at 95 °C and annealing/elongation for 1 min at 60 °C. Melting curves were generated after each run (from 60 °C to 95 °C, increasing 1°C/3 s).

Primer efficiencies were calculated for each assay based on three independent 5-fold dilution series made from a pool of all pre-amplified cDNA samples. C_q_ values were acquired using the Fluidigm Real-Time PCR Analysis software 3.0.2 (Fluidigm) and exported to GenEx5 (MultiD) for data processing including interplate calibration, correction for PCR efficiency, normalisation to mean expression of all miRNAs included in this study, and averaging of cDNA technical repeats.

### cDNA synthesis, pre-amplification, and qPCR of mRNA

Extracted total RNA was converted into cDNA by reverse transcription of 50 ng RNA using the QuantiTect Reverse Transcription Kit (Qiagen) as described previously^6^, including minus RT controls. cDNA was diluted 1:3 in low-EDTA TE-buffer (VWR – Bie & Berntsen) prior to 15 cycles of pre-amplification followed by exonuclease treatment as described previously^6^. Pre-amplified cDNA was diluted 1:10 in low-EDTA TE-buffer (VWR – Bie & Berntsen) for use in qPCR. A panel of immune genes were chosen for transcriptional analysis based on published findings. Subsequently, based on target identification of the miRNAs found to be significantly regulated, additional transcriptional analysis was performed for the identified genes. qPCR primers were designed using Primer3 (http://bioinfo.ut.ee/primer3-0.4.0/) as described previously^53^, and purchased from Sigma-Aldrich. Primer sequences and PCR efficiencies are shown in Supplementary Table S1.

qPCR was carried out in Dynamic Array Integrated Fluidic Circuit chips on the BioMark HD real-time PCR platform (Fluidigm) as described previously^6^. C_q_ values were acquired using the Fluidigm Real-Time PCR Analysis software 3.0.2 (Fluidigm) and exported to GenEx5 (MultiD) for data processing, including interplate correction, correction for PCR efficiency, normalisation to reference genes, and averaging of cDNA technical repeats. Using geNorm and NormFinder algorithms (in GenEx), peptidylprolyl isomerase A (*PPIA*), hypoxanthine phosphoribosyltransferase I (*HRPT1*), ribosomal protein L13a (*RPL13A*), TATA-box binding protein (*TBP*), and tyrosine 3-monooxygenase/tryptophan 5-monooxygenase activation protein, zeta polypeptide (*YWHAE*) where identified as the most stably expressed reference genes out of six candidates and used for mRNA data normalisation.

### Analysis for differential gene expression

For the initial screening study, normalised miRCURY LNA^TM^ data was log_2_ transformed before one-way ANOVA and Sidak correction (*p* < 0.0007) was employed to correct for multiple testing.

Normalised miRNA and mRNA qPCR expression data from Dynamic Array chips (Fluidigm) was log_2_ transformed and tested for normal distribution prior to Student’s *t*-test (or Mann-Whitney *U* test if normality of data could not be demonstrated). miRNA and mRNA expression in sample groups (group means) from time points after challenge (24 h, 72 h, and 14d pi) was compared to expression in samples from before challenge (group mean); fold changes of expression was determined by dividing the mean expression level of a post-challenge group with the mean expression of the pre-challenge group. A change in expression level was considered statistically and biologically significant if *p* < 0.05 and the relative gene expression difference was >1.5-fold (up or down) (miRNA) or >2-fold (up- or down) (mRNA) between the groups compared.

A clustered heatmap was constructed to visualise miRNA gene expression, using the HemI 1.0.2 Heatmap Illustrator Toolkit employing hierarchical clustering.

### miRNA target identification

Information on experimentally validated miRNA-target (i.e. mRNA) interactions (MTIs) was available from miRTarBase v. 6.0. An initial overview of potentially affected cellular pathways was gained from performing KEGG pathway enrichment analysis by submitting the list of target genes obtained from miRTarBase to DAVID Bioinformatics Resources v. 6.7 tool. The Benjamini-Hochberg procedure was applied to control the false discovery rate (*p* < 0.05). Pathway enrichment analysis was performed on four subsets of target genes: target genes for 1) miRNAs up-regulated at 24 h and 72 h pi (i.e. during active infection), 2) miRNAs down-regulated at 24 h and 72 h pi, 3) miRNAs up-regulated at 14d pi (i.e. after infection had cleared), and 4) miRNAs down-regulated at 14d pi.

MTIs for the sets of differentially expressed miRNAs in the three post-challenge groups were visualised using Cytoscape v. 3.2.1. This was achieved using the Cytoscape app CyTargetLinker v. 3.0.1; a CyTargetLinker RegIN containing only MTIs supported by strong experimental evidence as defined by miRTarBase was created, and applied for visualisation of MTI interaction networks in Cytoscape. KEGG pathway enrichment analysis and MTI networks were created using human miRNA and mRNA homologs, as miRTarBase does not contain porcine MTIs.

As available literature and miRNA databases are almost exclusively concerned with human miRNAs, a comparison of human and porcine miRNA sequences was made (miRBase v. 21) (summarised in Supplementary Table S2).

## Additional Information

**How to cite this article**: Brogaard, L. *et al*. Late regulation of immune genes and microRNAs in circulating leukocytes in a pig model of influenza A (H1N2) infection. *Sci. Rep.* **6**, 21812; doi: 10.1038/srep21812 (2016).

## Supplementary Material

Supplementary Information

## Figures and Tables

**Table 1:** Major differences and similarities of human, pig, and mouse disease signs, physiology, anatomy and immunology with regards to influenza A virus infection.

Symptom/characteristic	Human	Pig	Mouse	Reference
Fever	**Present**	**Present**	Absent	19,54
Nasal secretion	**Present**	**Present**	Absent	54
Coughing	**Present**	**Present**	Absent	19,21
Major sialic acid receptor of the upper respiratory tract	**α2-6-linked**	**α2-6-linked**	α2-3-linked	25,55,56
Possess tonsils	**Yes**	**Yes**	No	57
Nature of connective tissue	**Extensive and interlobular**	**Extensive and interlobular**	Little if any	26,27
Nature of pleurae	**Thick**	**Thick**	Thin	26
Early cytokine response to IAV infection		**Very similar to humans**	Somewhat similar to humans	6,20,23,24,28
Pulmonary intravascular macrophages	**Induced phagocytic cells**	Constitutive phagocytic cells	**Induced phagocytic cells**	58
Lymph node structure		Inverted	**As humans**	17
Interleukin 8	**Present**	**Present**	No direct homolog	59,60
Immunological reagents available	Many	Increasing	Many	30,31

**Table 2:** Relative expression levels of immune gene mRNA transcripts in porcine leukocytes before IAV challenge and at 24 h, 72 h, and 14d pi.

Gene	Before challenge (n = 12)		24 h pi (n = 12)			72 h pi (n = 9)			14d pi (n = 6)		
	Rel. expr. level	±95% CI	Rel. expr. level	±95% CI	*p*-value	Rel. expr. level	±95% CI	*p*-value	Rel. expr. level	±95% CI	*p*-value
*CASP1*	1.00	0.13	7.21	1.78	4E-12	2.18	0.58	0.00022	0.94	0.36	NS
*CASP3*	1.00	0.09	5.91	1.07	3E-13	1.36	0.35	NS	1.13	0.30	NS
CCL2*	1.00	0.41	2.34	0.50	0.0012	1.63	0.56	NS	1.53	0.70	NS
*CCL3*	1.00	0.24	0.59	0.22	0.030	1.07	0.70	NS	0.52	0.17	0.0067
*CD163*	1.00	0.47	3.58	1.50	0.00015	2.73	2.09	NS	0.48	0.18	0.047
*CXCL2*	1.00	0.35	0.96	0.18	NS	0.99	0.78	NS	0.39	0.16	0.019
CXCL10*	1.00	0.33	283	92.56	7E-16	5.84	4.39	0.011	3.43	5.81	NS
DDX58*	1.00	0.23	13.01	2.01	6E-13	1.53	0.66	NS	0.64	0.42	NS
*FAS*	1.00	0.11	5.52	1.02	4E-12	0.94	0.24	NS	0.73	0.19	0.018
*FASLG*	1.00	0.39	2.42	0.62	0.026	1.06	0.57	NS	0.65	0.23	NS
*IDO1*	1.00	0.30	133	98.20	8E-09	0.71	0.28	NS	0.55	0.27	0.035
*IFITM1*	1.00	0.27	5.66	0.98	2E-10	1.62	0.49	0.040	0.81	0.24	NS
*IFITM3*	1.00	0.40	3.93	0.69	7E-8	1.56	0.54	NS	0.78	0.27	NS
*IFNA1*	1.00	0.26	2.86	1.32	0.0022	0.72	0.23	NS	1.31	0.59	NS
*IL8*	1.00	0.32	1.86	0.60	0.018	1.37	0.44	NS	1.17	0.20	NS
*IL10*	1.00	0.39	4.49	1.04	2E-06	1.55	0.40	NS	0.93	0.23	NS
*IL18*	1.00	0.28	0.09	0.04	3E-08	0.60	0.52	0.020	0.50	0.28	0.028
*IL1RAP*	1.00	0.34	9.24	2.32	8E-09	0.70	0.32	NS	0.41	0.22	0.014
*IL1RN*	1.00	0.27	18.14	3.56	3E-14	1.10	0.29	NS	0.97	0.23	NS
*IRF1*	1.00	0.19	1.80	0.31	0.00030	0.57	0.13	0.0015	0.75	0.26	NS
*IRF2*	1.00	0.16	4.66	0.81	2E-10	1.09	0.25	NS	0.78	0.19	NS
*IRF3*	1.00	0.21	2.45	0.48	3E-06	0.99	0.18	NS	0.66	0.15	0.033
*IRF9*	1.00	0.25	1.88	0.25	0.00011	0.95	0.17	NS	0.72	0.22	NS
IFIH1*	1.00	0.20	5.37	0.73	3E-11	1.15	0.22	NS	0.80	0.30	NS
*JAK2*	1.00	0.17	3.79	0.88	2E-8	1.35	0.30	NS	1.10	0.36	NS
*MCL1*	1.00	0.20	2.06	0.26	4E-06	0.90	0.17	NS	0.82	0.13	NS
MX1*	1.00	0.23	13.72	2.09	1E-13	2.10	0.70	0.0018	0.87	0.65	NS
*MYD88*	1.00	0.28	1.58	0.39	0.031	0.90	0.44	NS	0.61	0.23	NS
*NOD1*	1.00	0.23	2.55	0.36	7E-5	1.08	0.25	NS	0.74	0.09	NS
OASL*	1.00	0.26	20.40	4.14	7E-12	2.11	1.06	NS	1.20	1.10	NS
*PTGS2*	1.00	0.26	1.33	0.28	NS	0.87	0.23	NS	0.39	0.12	0.0012
STAT1*	1.00	0.09	4.13	0.46	8E-14	1.11	0.25	NS	0.88	0.25	NS
*TICAM1*	1.00	0.25	2.47	0.66	0.00016	1.47	0.43	NS	1.08	0.29	NS
*TICAM2*	1.00	0.19	3.30	0.69	2E-08	0.84	0.14	NS	0.58	0.11	0.0017
*TLR2*	1.00	0.34	2.30	0.45	0.00035	1.12	0.52	NS	0.81	0.32	NS
*TLR3*	1.00	0.23	2.42	0.48	4E-05	1.40	0.35	NS	0.51	0.12	0.0078
*TLR4*	1.00	0.26	8.41	2.90	8E-09	1.32	0.64	NS	0.56	0.26	0.023
*TLR7*	1.00	0.14	4.23	0.51	4E-12	1.34	0.34	NS	1.09	0.34	NS
*TLR8*	1.00	0.22	2.17	0.59	0.00014	1.77	0.34	0.00065	1.11	0.22	NS
*TNF*	1.00	0.21	0.55	0.15	0.0027	0.79	0.42	NS	0.35	0.11	0.00024

Genes included are all statistically significantly >2-fold up- or down-regulated at at least one time point after challenge (*p* < 0.05); NS: not significant. Genes marked with * have previously been reported as classifiers for human IAV infection (H1N1, H3N2)^33^,^34^,^35^,^36^,^37^.
