# Supplementary material for: Late regulation of immune genes and microRNAs in circulating leukocytes in a pig model of influenza A (H1N2) infection
Source: Sci Rep. 2016 Feb 19;6:21812. doi: 10.1038/srep21812 (PMC4759598; doi:10.1038/srep21812)
Supplement: Supplementary Information [file srep21812-s1.pdf]

Supplementary material for:

Late regulation of immune genes and microRNAs in circulating leukocytes in a pig model of influenza A (H1N2) infection

Louise Brogaard, Peter M. H. Heegaard, Lars E. Larsen, Shila Mortensen, Michael Schlegel, Ralf Dürrwald, Kerstin Skovgaard

Supplementary Table S1. qPCR primer sequences and experimentally determined PCR efficiencies for all reported genes and miRNAs in the present study.

Gene/miRNA	Forward primer	Reverse primer	PCR eff.
CASP1	GAAGGACAAACCCAAGGTGA	TGGGCTTTCTTAATGGCATC	101 %
CASP3	AGCAGTTTTATTTGCGTGCTT	CAACAGGTCCATTTGTTCCA	99 %
CASP9	CTGTGAGGACCTGCTGACC	CGGAGGAAATTAAAACAGCCAGG	104 %
CCL2	CTTCTGCACCCAGGTCCTT	CGCTGCATCGAGATCTTCTT	90 %
CCL3	CCAGGTCTTCTCTGCACCAC	GCTACGAATTTGCGAGGAAG	104 %
CD163	CACATGTGCCAACAAAATAAGAC	CACCACCTGAGCATCTTCAA	117 %
CXCL2	GAAGATGCTAAACAAGAGCAGTG	AGCCAAATGCATGAAACACA	112 %
CXCL10	CCCACATGTTGAGATCATTGC	GCTTCTCTCTGTGTTCGAGGA	97 %
DDX58	ACGAAAGGGGAAGGTTGTCT	ATGCCTGCAACTTTGTACCC	96 %
FAS	CACTGTAACCCTTGCACCAC	TGGAACACTTCTCTGCATTTGG	109 %
FASLG	TTCTGGTGGCCCTGGTTG	CTTTGGCTGGCAGACTCTCT	106 %
IDO1	GGGCCCATGACTTACAAGAA	TTTCCACCAATAGCGAAACC	97 %
IFITM1	GCTTTCGCCTACTCCGTGA	CCAGGATCAGAGCCCAGATG	106 %
IFITM3	TGAACTGCGCTTCCCAGC	CACCTCGTGCTCCTCCTTG	104 %
IFNA1	TTCCAGCTCTTCAGCACAGA	AGCTGCTGATCCAGTCCAGT	101 %
IFNG	CCATTCAAAGGAGCATGGAT	TTCAGTTTCCCAGAGCTACCA	94 %
IL8	TTGCCAGAGAAATCACAGGA	TGCATGGGACACTGGAAATA	89 %
IL10	TACAACAGGGGCTTGCTCTT	GCCAGGAAGATCAGGCAATA	108 %
IL18	CAATTGCATCAGCTTTGTGG	TCCAGGTCCTCATCGTTTTC	100 %
IL1RAP	TGCATCTTTGACCGAGACAG	GGGCTCAGGACAACAATCAT	93 %
IL1RN	TGCCTGTCCTGTGTCAAGTC	GTCCTGCTCGCTGTTCTTTC	103 %
IRF1	TGAAGCTGCAACAGATGAGG	CTTCCCATCCACGTTTGTCT	102 %
IRF2	GATGCTGCCCTTATCTGAGC	TGTGCTTCACTCTGTCTTCCTT	91 %
IRF3	GCTACACCCTCTGGTTCTGC	GAGACACATGGGGACAACCT	96 %
IRF9	CATTCAGACTTGGGGAGCAG	AAAGGGGCCTCAGTGGTAAC	101 %
IFIH1	CAGTGTGCTAGCCTGCTCTG	GCAGTGCCTTGTTTCCTCTC	97 %
JAK2	CTCAGATATGCAAGGGTATGGAGT	CCACCAATATATTCCTTGTTGCCA	104 %
MCL1	GAGGCTGGGATGGGTTTGTG	TGCCAAACCAGCTCCTACTC	103 %
MX1	CCTCCACAGAACTGCCAAG	GCAGTACACGATCTGCTCCA	98 %
MYD88	AGCTGTAGGGGGAATGTGTG	TCAGCTGGTCTGTGGATGTG	98 %
NOD1	CTCGACCTGGACAACAACAA	TGAGTCTGATGACCGTGAGG	105 %
OASL	TGGTACCTGAAGTACGTGAAAGC	TACCCACTTCCCAGGCATAG	90 %
PTGS2	AGGCTGATACTGATAGGAGAAACG	GCAGCTCTGGGTCAAACTTC	96 %
STAT1	CCTTGCAGAATAGAGAACATGATAC	CCTTTCTCTTGTTGTCAAGCATT	98 %
TICAM1	CTGCCTTCCCACAGCCTC	AGCCCCAGTTGTACCATTTGA	90 %
TICAM2	TCTGCTGCAAAATGACTTCGG	AGCCATTGACAGCATCGTCT	90 %
TLR2	GTTTTACGGAAATTGTGAAACTG	TCCACATTACCGAGGGATTT	92 %
TLR3	ATTGTGCAAAAGATTCAAGGTG	TCTTCGCAAACAGAGTGCAT	104 %
TLR4	TTTCCACAAAAGTCGGAAGG	CAACTTCTGCAGGACGATGA	95 %
TLR7	GGAAATAGCATCAGCCAAGCTC	TTCCAGGTTGCGTAGCTCTT	87 %
TLR8	GCAAAGACCACCACCAAC	ATCCGTCAGTCTGGGAAT	95 %
TNF	CCCCCAGAAGGAAGAGTTTC	CGGGCTTATCTGAGGTTTGA	94 %
Genes identified as targeted in MTI network analysis			
AKT2	CTGCTTAAGAAGGACCCAAAGC	CTTCTGTACCACGTCCTGCC	95 %
BCL2	GACTCCCTTCACCGCGAG	CTCTCCACACACATGACCCC	90 %
CDK2	TTGCTGAGATGGTGACCCG	GGGTCCCAAGAGTCCGAAAG	106 %
CDK4	GGCCAGAATCTACAGCTACCAG	TCCACAGGTGTTGCATACGT	90 %

CCNE2	ATGGTGCTTGCAGTGAAGAGG	TGGAGGAAGAGATTTAGCCAGG	107 %
CXCR4	CTGCTGGCTGCCATACTACA	TCAAACTCACACCCTTGCTG	97 %
FOS	GGAACAGTTGTCCCCAGAAG	TGTCAGTCAGCTCCCTCCTC	100 %
FOXO3A	CCAGTCTATGCAAACCCTCTCG	CAAGTCGCTGGGGAACTTCT	90 %
PTEN	AGCAAATAAAGACAAGGCCAACC	GTTGAACTGCTAGCCTCTGGA	103 %
SP1	AAGATAGTGAAGGAAGGGGCTC	TACTTTGCCACAACCTTGCATG	106 %
TP53	TAAGCGAGCACTGCCCAC	TCTCGGAACATCTCGAAGCG	101 %
VEGFA	CGAAGGTCTGGAGTGTGTGC	TCTCTCCTATGTGCTGGCCT	90 %
hsa-miR-223-5p	GCGTGTATTTGACAAGCTG	GTCCAGTTTTTTTTTTTTTTTTAACTCAG	97 %
ssc-miR-182	AGTTTGGCAATGGTAGAACTC	GTCCAGTTTTTTTTTTTTTTTTAGTGTG	96 %
ssc-miR-29a	GCTAGCACCATCTGAAATCG	TCCAGTTTTTTTTTTTTTTTTAACCGA	100 %
ssc-miR-31	GGCAAGATGCTGGCA	CCAGTTTTTTTTTTTTTTTTCAGCTATG	92 %
ssc-miR-29b	CAGTAGCACCATTTGAAATCAG	GGTCCAGTTTTTTTTTTTTTTTTAACACT	96 %
hsa-miR-203a-3p	AGGTGAAATGTTTAGGACCAC	GTCCAGTTTTTTTTTTTTTTTTCTAGTG	99 %
hsa-miR-449a	AGTGGCAGTGTATTGTTAGC	GTCCAGTTTTTTTTTTTTTTTTACCAG	90 %
ssc-miR-21	TCAGTAGCTTATCAGACTGATG	CGTCCAGTTTTTTTTTTTTTTTTCAAC	101 %
hsa-miR-23a-3p	CATCACATTGCCAGGGAT	CGTCCAGTTTTTTTTTTTTTTTTGGAA	109 %
ssc-miR-23b	AGATCACATTGCCAGGGA	CCAGTTTTTTTTTTTTTTTTGGTAATCC	104 %
ssc-miR-30c-5p	CAGTGTAAACATCCTACACTCTC	CCAGTTTTTTTTTTTTTTTTGCTGAG	109 %
ssc-miR-423-5p	GGGCAGAGAGCGAGAC	GGTCCAGTTTTTTTTTTTTTTTTAAAGTC	90 %
hsa-miR-150-5p	GTCTCCCAACCCTTGTAC	GTCCAGTTTTTTTTTTTTTTTTCACTG	115 %
ssc-miR-15a	CAGTAGCAGCACATAATGGT	TCCAGTTTTTTTTTTTTTTTTACAAACC	97 %
ssc-miR-186	CGCAGCAAAGAATTCTCCT	GGTCCAGTTTTTTTTTTTTTTTTAAGC	102 %
ssc-miR-22-5p	CAGAGTTCTTCAGTGGCAAG	GGTCCAGTTTTTTTTTTTTTTTTAAAGC	90 %
ssc-miR-28-5p	CAGAAGGAGCTCACAGTCT	GGTCCAGTTTTTTTTTTTTTTTTCTCA	97 %
ssc-miR-146a-5p	GCAGTGAGAACTGAATTCCA	GGTCCAGTTTTTTTTTTTTTTTTAACC	92 %
hsa-miR-223-3p	CGCAGTGTCAGTTTGTCA	CCAGTTTTTTTTTTTTTTTTGGGGTA	101 %
hsa-miR-16-5p	GCAGTAGCAGCACGTA	CAGTTTTTTTTTTTTTTTTCGCCAA	96 %

Supplementary Table S2. Sequence comparison of human and porcine homologs of the miRNAs reported regulated in the present study. Underlined nucleotides: nucleotides 2-7, i.e. the seed sequence.

miRBase accession number	miRNA	miRNA sequence
MIMAT0000086	hsa-miR-29a-3p	uagcaccaucugaaaucgguua
MIMAT0013870	ssc-miR-29a	cuagcaccaucugaaaucgguua
MIMAT0000089	hsa-miR-31-5p	aggcaagaugcuggcauagcu
MIMAT0025360	ssc-miR-31	aggcaagaugcuggcauagcug
MIMAT0000100	hsa-miR-29b-3p	uagcaccauuugaaaucaguguu
MIMAT0002137	ssc-miR-29b	uagcaccauuugaaaucaguguu
MIMAT0000076	hsa-miR-21-5p	uagcuuaucagacugauguuga
MIMAT0002165	ssc-miR-21	uagcuuaucagacugauguuga
MIMAT0000078	hsa-miR-23a-3p	aucacauugccagggauuucc
MIMAT0002133	ssc-miR-23a	aucacauugccagggauuucc
MIMAT0000244	hsa-miR-30c-5p	uguaaacauccuacacucucagc
MIMAT0002167	ssc-miR-30c-5p	uguaaacauccuacacucucagc
MIMAT0004748	hsa-miR-423-5p	ugaggggcagagagcgagacuuu
MIMAT0013880	ssc-miR-423-5p	ugaggggcagagagcgagacuuu
MIMAT0000451	hsa-miR-150-5p	ucucccaacccuuguaccagug
MIMAT0025365	ssc-miR-150	ucucccaacccuuguaccagug
MIMAT0000456	hsa-miR-186-5p	caaagaauucuccuuuugggcu
MIMAT0002162	ssc-miR-186	caaagaauucuccuuuugggcuu
MIMAT0004495	hsa-miR-22-5p	aguucuucaguggcaagcuuua
MIMAT0015709	ssc-miR-22-5p	aguucuucaguggcaagcuuua
MIMAT0000085	hsa-miR-28-5p	aaggagcucacagucuauugag
MIMAT0002136	ssc-miR-28-5p	aaggagcucacagucuauugag
MIMAT0000449	hsa-miR-146a-5p	ugagaacugaauuccauggguu
MIMAT0022963	ssc-miR-146a-5p	ugagaacugaauuccaugggu u
MIMAT0000069	hsa-miR-16-5p	uagcagcacguaaauauuggcg
MIMAT0007754	ssc-miR-16	uagcagcacguaaauauuggcg
MIMAT0000259	hsa-miR-182-5p	uuuggcaaugguagaacucacacu
MIMAT0025366	ssc-miR-182	uuuggcaaugguagaacucacacu
MIMAT0000418	hsa-miR-23b-3p	aucacauugccagggauuacc
MIMAT0013893	ssc-miR-23b	aucacauugccagggauuacca
MIMAT0000068	hsa-miR-15a-5p	uagcagcacauaaugguuugug
MIMAT0007753	ssc-miR-15a	uagcagcacauaaugguuugu

Supplementary Table S3. Expression levels of miRNA in porcine leukocytes after IAV challenge.

Expression is shown as relative levels compared to before challenge.

miRNA	Rel. expression level	±95 % CI	*p*-value
24h pi (n = 12)			
hsa-miR-223-5p	1.94	0.41	0.00030
ssc-miR-182	1.70	0.47	0.020
ssc-miR-29a	0.65	0.18	0.028
ssc-miR-31	0.45	0.28	0.0027
72h pi (n = 9)			
ssc-miR-29b	2.16	0.46	0.000048
hsa-miR-203a-3p	1.82	0.28	0.00024
hsa-miR-449a	1.80	0.74	0.038
ssc-miR-21	1.60	0.47	0.033
ssc-miR-29a	1.55	0.36	0.0071
hsa-miR-23a-3p	0.63	0.18	0.036
ssc-miR-23b	0.60	0.16	0.024
ssc-miR-30c-5p	0.59	0.10	0.0074
ssc-miR-423-5p	0.53	0.11	0.011
hsa-miR-150-5p	0.52	0.15	0.042
14d pi (n = 6)			
ssc-miR-15a	2.31	0.98	0.022
ssc-miR-29b	2.30	0.78	0.0057
ssc-miR-29a	2.29	0.76	0.0032
hsa-miR-449a	2.05	0.82	0.028
ssc-miR-186	1.84	0.52	0.010
ssc-miR-22-5p	1.64	0.47	0.013
ssc-miR-28-5p	1.60	0.47	0.0076
hsa-miR-203a-3p	1.56	0.31	0.010
ssc-miR-146a-5p	1.52	0.24	0.010
hsa-miR-150-5p	0.50	0.21	0.022
ssc-miR-23b	0.44	0.15	0.017
hsa-miR-223-3p	0.42	0.15	0.010
hsa-miR-23a-3p	0.42	0.14	0.0076
hsa-miR-16-5p	0.35	0.26	0.022

Supplementary Table S4. KEGG Pathway enrichment analysis of experimentally validated targets of the miRNAs found to be differentially expressed in the present study. Pathways highlighted in bold text are enriched in at least three of the four gene subsets. Many pathways related to specific cancer types were also enriched in all four gene subsets; these have not been included in the table.

KEGG pathway	Number of genes involved	Percentage of investigated genes involved	P-Value	Benjamini-Hochberg adjusted P-Value
1) Enriched in target genes for up-regulated miRNAs at 24h and 72h pi				
**Pathways in cancer**	55	19,3	1,60E-27	1,80E-25
**Focal adhesion**	36	12,6	2,30E-18	8,50E-17
**Cell cycle**	20	7	3,20E-09	3,50E-08
**p53 signaling pathway**	15	5,3	7,70E-09	7,70E-08
**MAPK signaling pathway**	26	9,1	2,30E-07	1,80E-06
**Apoptosis**	13	4,6	8,40E-06	5,50E-05
**Neurotrophin signaling pathway**	15	5,3	1,60E-05	9,70E-05
**Cytokine-cytokine receptor interaction**	22	7,7	2,70E-05	1,60E-04
**ErbB signaling pathway**	12	4,2	4,70E-05	2,60E-04
TGF-beta signaling pathway	12	4,2	4,70E-05	2,60E-04
T cell receptor signaling pathway	13	4,6	7,70E-05	4,00E-04
**Adherens junction**	11	3,9	8,30E-05	4,20E-04
**ECM-receptor interaction**	11	3,9	1,70E-04	8,40E-04
Toll-like receptor signaling pathway	12	4,2	1,90E-04	8,50E-04
Regulation of actin cytoskeleton	17	6	6,10E-04	2,70E-03
**Progesterone-mediated oocyte maturation**	10	3,5	9,70E-04	4,10E-03
Gap junction	10	3,5	1,20E-03	5,00E-03
Dilated cardiomyopathy	10	3,5	1,60E-03	5,90E-03
**Jak-STAT signaling pathway**	13	4,6	2,10E-03	7,80E-03
**GnRH signaling pathway**	10	3,5	2,40E-03	8,60E-03
Hypertrophic cardiomyopathy (HCM)	9	3,2	3,60E-03	1,20E-02
B cell receptor signaling pathway	8	2,8	6,70E-03	2,20E-02
**VEGF signaling pathway**	8	2,8	6,70E-03	2,20E-02
Fc gamma R-mediated phagocytosis	9	3,2	7,10E-03	2,30E-02
2) Enriched in target genes for down-regulated miRNAs at 24h and 72h pi				
**Pathways in cancer**	37	19,1	8,60E-16	9,70E-14
**p53 signaling pathway**	14	7,2	3,80E-09	1,40E-07
**Focal adhesion**	22	11,3	5,50E-09	1,50E-07
**Tight junction**	12	6,2	2,80E-04	2,20E-03
**Adherens junction**	9	4,6	4,10E-04	2,80E-03
**Cell cycle**	11	5,7	6,60E-04	4,20E-03
**ECM-receptor interaction**	9	4,6	7,40E-04	4,50E-03

Notch signaling pathway	7	3,6	7,40E-04	4,20E-03
**Apoptosis**	9	4,6	9,40E-04	5,10E-03
**Jak-STAT signaling pathway**	11	5,7	3,40E-03	1,70E-02
**Chemokine signaling pathway**	12	6,2	4,40E-03	2,10E-02
Epithelial cell signaling in Helicobacter pylori infection	7	3,6	5,10E-03	2,40E-02
Viral myocarditis	7	3,6	6,30E-03	2,80E-02
Cysteine and methionine metabolism	5	2,6	7,90E-03	3,40E-02
**Neurotrophin signaling pathway**	9	4,6	8,60E-03	3,50E-02
**Wnt signaling pathway**	10	5,2	8,90E-03	3,60E-02
3) Enriched in target genes for up-regulated miRNAs at 14d pi				
**Pathways in cancer**	63	24,8	8,80E-37	9,50E-35
**Focal adhesion**	39	15,4	8,10E-22	2,90E-20
**Cell cycle**	25	9,8	4,90E-14	5,30E-13
Toll-like receptor signaling pathway	22	8,7	4,20E-13	4,10E-12
**Neurotrophin signaling pathway**	21	8,3	2,10E-10	1,60E-09
**MAPK signaling pathway**	28	11	6,30E-09	4,00E-08
**Apoptosis**	16	6,3	1,80E-08	1,10E-07
**p53 signaling pathway**	14	5,5	4,60E-08	2,60E-07
T cell receptor signaling pathway	17	6,7	5,50E-08	3,00E-07
**ErbB signaling pathway**	15	5,9	1,30E-07	6,90E-07
**Chemokine signaling pathway**	21	8,3	3,00E-07	1,50E-06
B cell receptor signaling pathway	13	5,1	1,20E-06	5,60E-06
**Adherens junction**	13	5,1	1,60E-06	7,10E-06
Fc gamma R-mediated phagocytosis	14	5,5	2,60E-06	1,10E-05
**VEGF signaling pathway**	12	4,7	8,10E-06	3,20E-05
Regulation of actin cytoskeleton	20	7,9	1,10E-05	4,10E-05
**Cytokine-cytokine receptor interaction**	22	8,7	1,60E-05	6,10E-05
**Progesterone-mediated oocyte maturation**	12	4,7	3,10E-05	1,10E-04
**Wnt signaling pathway**	15	5,9	1,00E-04	3,50E-04
Epithelial cell signaling in Helicobacter pylori infection	10	3,9	1,30E-04	4,30E-04
Leukocyte transendothelial migration	13	5,1	1,30E-04	4,30E-04
Fc epsilon RI signaling pathway	10	3,9	3,70E-04	1,20E-03
**ECM-receptor interaction**	10	3,9	6,40E-04	2,00E-03
mTOR signaling pathway	8	3,1	6,60E-04	2,00E-03
TGF-beta signaling pathway	10	3,9	8,30E-04	2,40E-03
Gap junction	10	3,9	9,80E-04	2,80E-03
Axon guidance	12	4,7	1,20E-03	3,20E-03
**Jak-STAT signaling pathway**	13	5,1	1,60E-03	4,20E-03
NOD-like receptor signaling pathway	8	3,1	1,90E-03	4,90E-03
Type II diabetes mellitus	7	2,8	2,10E-03	5,30E-03
Insulin signaling pathway	11	4,3	5,40E-03	1,30E-02
**GnRH signaling pathway**	9	3,5	7,10E-03	1,70E-02
Hypertrophic cardiomyopathy (HCM)	8	3,1	1,10E-02	2,60E-02

Intestinal immune network for IgA production	6	2,4	1,30E-02	2,90E-02
Oocyte meiosis	9	3,5	1,40E-02	3,10E-02
Natural killer cell mediated cytotoxicity	10	3,9	1,40E-02	3,10E-02
**Tight junction**	10	3,9	1,50E-02	3,20E-02
RIG-I-like receptor signaling pathway	7	2,8	1,60E-02	3,40E-02
Dilated cardiomyopathy	8	3,1	1,70E-02	3,40E-02
Endocytosis	12	4,7	1,70E-02	3,40E-02
Prion diseases	5	2	1,80E-02	3,50E-02
4) Enriched in target genes for down-regulated miRNAs at 14d pi				
**Pathways in cancer**	35	23,6	1,40E-16	1,20E-14
**p53 signaling pathway**	12	8,1	7,30E-08	2,60E-06
**Jak-STAT signaling pathway**	14	9,5	1,10E-05	1,10E-04
**Cell cycle**	12	8,1	3,40E-05	3,00E-04
**Cytokine-cytokine receptor interaction**	16	10,8	2,00E-04	1,20E-03
Insulin signaling pathway	11	7,4	3,30E-04	2,00E-03
**Focal adhesion**	13	8,8	6,20E-04	3,50E-03
**Chemokine signaling pathway**	12	8,1	1,20E-03	6,30E-03
NOD-like receptor signaling pathway	7	4,7	1,40E-03	7,10E-03
Dorso-ventral axis formation	5	3,4	1,40E-03	6,80E-03
**ErbB signaling pathway**	8	5,4	1,70E-03	7,70E-03
**Apoptosis**	8	5,4	1,70E-03	7,70E-03
Adipocytokine signaling pathway	7	4,7	2,10E-03	9,30E-03
Notch signaling pathway	6	4,1	2,30E-03	9,90E-03
**Neurotrophin signaling pathway**	9	6,1	3,20E-03	1,30E-02
**VEGF signaling pathway**	7	4,7	3,70E-03	1,50E-02
**Tight junction**	9	6,1	5,20E-03	2,00E-02
**MAPK signaling pathway**	13	8,8	6,70E-03	2,50E-02
**Progesterone-mediated oocyte maturation**	7	4,7	7,20E-03	2,60E-02
**Wnt signaling pathway**	9	6,1	1,00E-02	3,50E-02

Supplementary Figure S1. miRNA-target interaction networks at A) 24h pi, B) 72h pi, and c) 14d pi. Red circles represent down-regulated miRNAs; green circles represent up-regulated miRNAs; hexagons represent experimentally validated gene targets. Hexagons with solid grey fill are genes that interact with two or more of the regulated miRNAs. Experimentally validated interactions are indicated with arrows from miRNA to target. The number of genes with two or more experimentally validated MTIs comprised 6, 65, and 84 at 24h, 72h, and 14d pi, respectively.

a) 24h pi

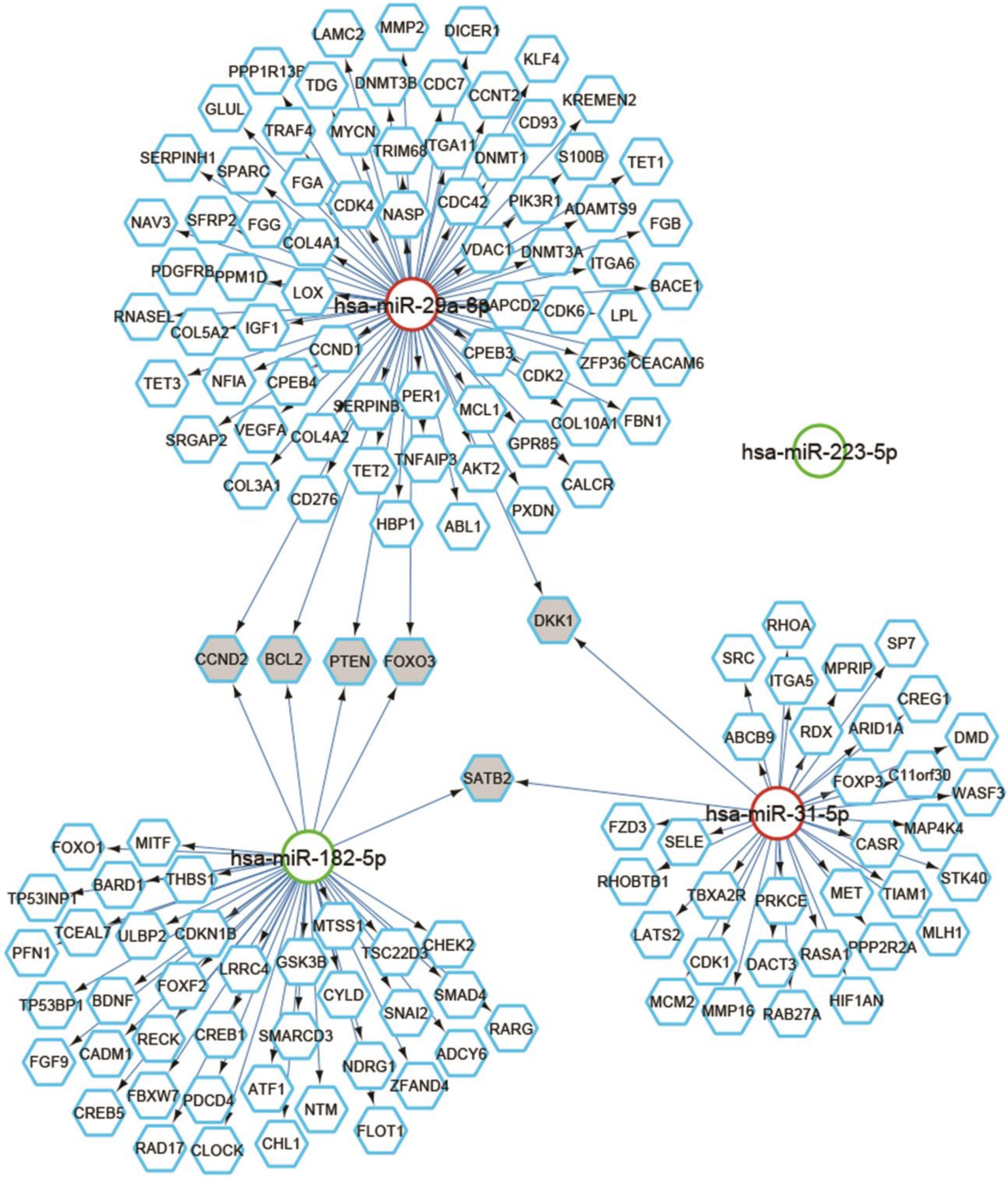
hsa-miR-29a-3p
hsa-miR-223-5p
hsa-miR-182-5p
hsa-miR-31-5p
LAMC2
MMP2
DICER1
KLF4
PPP1R13B
TDG
DNMT3B
CDC7
CCNT2
KREMEN2
GLUL
TRAF4
MYCN
TRIM68
ITGA11
CD93
SERPINH1
SPARC
FGA
DNMT1
S100B
TET1
CDK4
NASP
CDC42
PIK3R1
ADAMTS9
NAV3
SFRP2
FGG
COL4A1
VDAC1
DNMT3A
FGB
ITGA6
PDGFRB
PPM1D
LOX
BACE1
RNASEL
COL5A2
IGF1
APCD2
CDK6
LPL
CCND1
CPEB3
ZFP36
CEACAM6
TET3
NFIA
CPEB4
CDK2
SERPINB
PER1
MCL1
COL10A1
FBN1
SRGAP2
VEGFA
COL4A2
GPR85
TNFAIP3
COL3A1
TET2
AKT2
CALCR
CD276
PXDN
HBP1
ABL1
DKK1
CCND2
BCL2
PTEN
FOXO3
SATB2
RHOA
SRC
SP7
ITGA5
MPRIP
CREG1
ABCB9
RDX
ARID1A
DMD
FOXP3
C11orf30
WASF3
FZD3
SELE
CASR
MAP4K4
RHOBTB1
TBXA2R
PRKCE
MET
TIAM1
STK40
LATS2
MLH1
CDK1
DACT3
RASA1
PPP2R2A
MCM2
MMP16
RAB27A
HIF1AN
FOXO1
MITF
BARD1
THBS1
TP53INP1
TCEAL7
ULBP2
CDKN1B
MTSS1
CHEK2
PFN1
LRRC4
GSK3B
TSC22D3
TP53BP1
BDNF
FOXF2
CYLD
SMAD4
SNAI2
RARG
FGF9
CADM1
RECK
CREB1
SMARCD3
ADCY6
NDRG1
ZFAND4
CREB5
FBXW7
PDCD4
ATF1
NTM
FLOT1
RAD17
CLOCK
CHL1

b) 72h pi

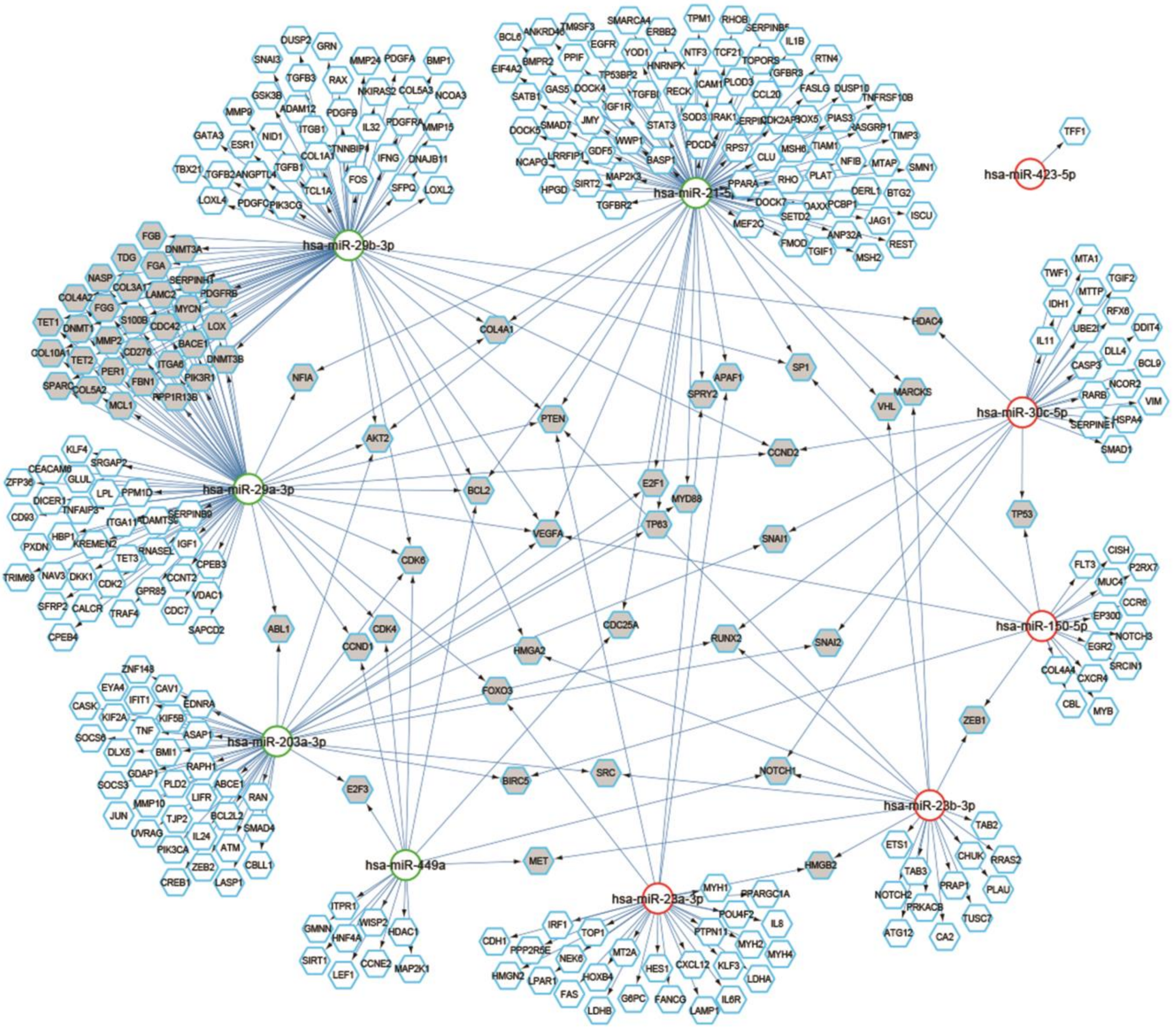

hsa-miR-29b-3p
hsa-miR-29a-3p
hsa-miR-203a-3p
hsa-miR-449a
hsa-miR-23a-3p
hsa-miR-23b-3p
hsa-miR-150-5p
hsa-miR-30c-5p
hsa-miR-423-5p

c) 14d pi

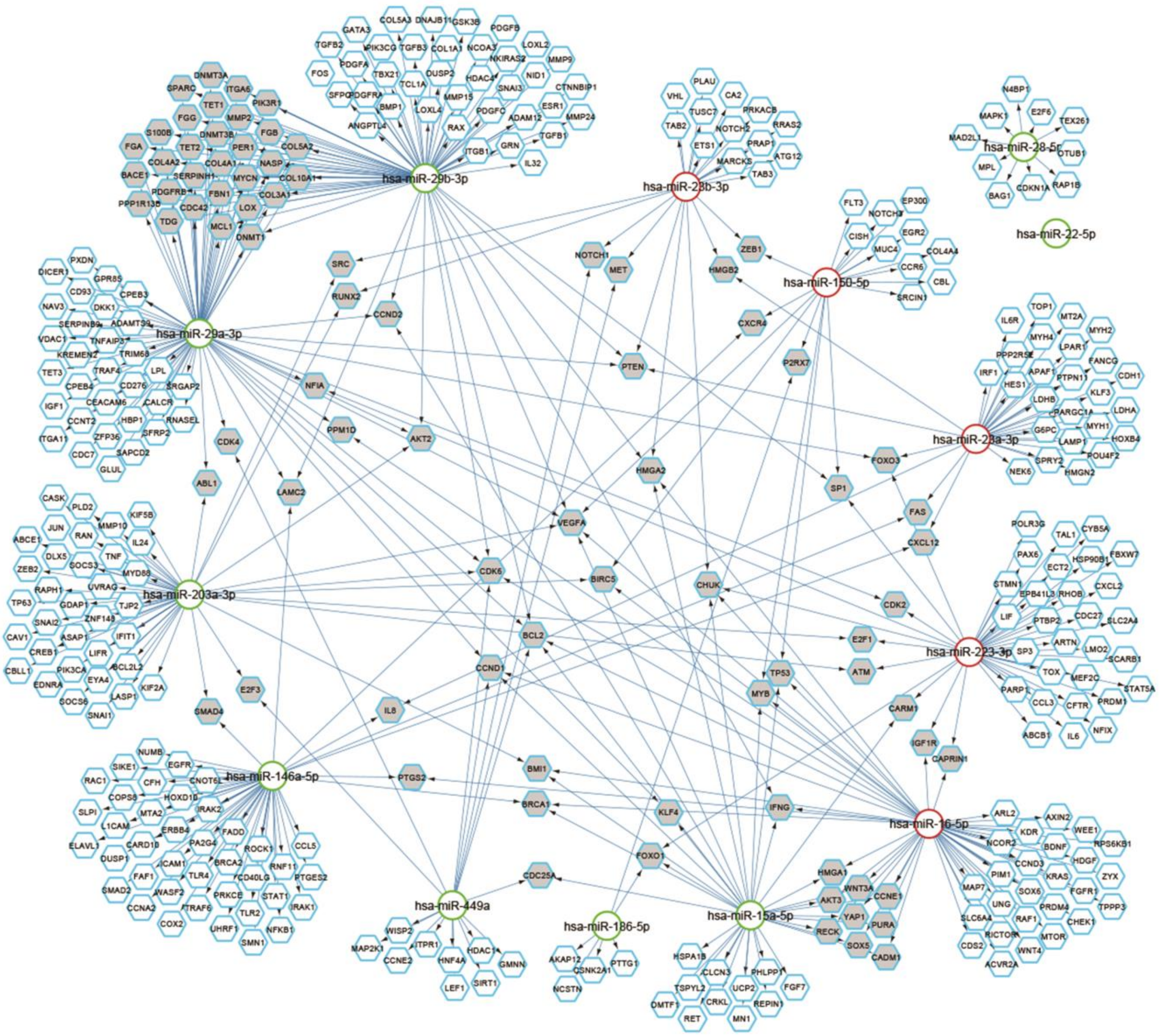
hsa-miR-29b-3p
hsa-miR-23b-3p
hsa-miR-28-5p
hsa-miR-22-5p
hsa-miR-150-5p
hsa-miR-29a-3p
hsa-miR-23a-3p
hsa-miR-203a-3p
hsa-miR-223-3p
hsa-miR-146a-5p
hsa-miR-16-5p
hsa-miR-449a
hsa-miR-186-5p
hsa-miR-15a-5p
